# The Absence of N-Acetyl-D-glucosamine Causes Attenuation of Virulence of *Candida albicans* upon Interaction with Vaginal Epithelial Cells *In Vitro*

**DOI:** 10.1155/2015/398045

**Published:** 2015-08-20

**Authors:** Máté Manczinger, Alexandra Bocsik, Gabriella F. Kocsis, Andrea Vörös, Zoltán Hegedűs, Lilla Ördögh, Éva Kondorosi, Annamária Marton, Csaba Vízler, Vilmos Tubak, Mária Deli, Lajos Kemény, István Nagy, Lóránt Lakatos

**Affiliations:** ^1^Department of Dermatology and Allergology, University of Szeged, Szeged, Hungary; ^2^MTA-SZTE Dermatological Research Group, Hungary; ^3^Institute of Biophysics, Biological Research Centre of the Hungarian Academy of Sciences, Szeged, Hungary; ^4^Institute of Biochemistry, Biological Research Centre of the Hungarian Academy of Sciences, Szeged, Hungary; ^5^Creative Laboratory Ltd., Szeged, Hungary

## Abstract

To better understand the molecular events underlying vulvovaginal candidiasis, we established an *in vitro* system. Immortalized vaginal epithelial cells were infected with live, yeast form *C. albicans* and *C. albicans* cultured in the same medium without vaginal epithelial cells were used as control. In both cases a yeast to hyphae transition was robustly induced. Whole transcriptome sequencing was used to identify specific gene expression changes in *C. albicans*. Numerous genes leading to a yeast to hyphae transition and hyphae specific genes were upregulated in the control hyphae and the hyphae in response to vaginal epithelial cells. Strikingly, the GlcNAc pathway was exclusively triggered by vaginal epithelial cells. Functional analysis in our *in vitro* system revealed that the GlcNAc biosynthesis is involved in the adherence to, and the ability to kill, vaginal epithelial cells *in vitro*, thus indicating the key role for this pathway in the virulence of *C. albicans* upon vulvovaginal candidiasis.

## 1. Introduction

*Candida albicans* is an opportunistic pathogen whose invasion correlates with changes in environmental factors such as alterations to host immunity, competition with other saprophytes, and physical perturbation of its niche. Many consider* C. albicans* to be obligately associated with mammalian hosts; clearly a key for understanding the pathogenicity of this fungus lies in the regulatory processes that determine its transition from a commensal to a pathogen.

*C. albicans* is a dimorphic yeast and one of the most common members of the human commensal flora [1]. The yeast form colonizes mucosal surfaces of the oral cavity, gastro-intestinal and reproductive tracts, and the skin [2]. However, under some host conditions* C. albicans* can undergo a morphological transition and can become pathogenic. The developmental transition from the predominant yeast form to the hyphal form of* C. albicans* is considered an early step in the invasion of epithelial tissues; however both forms can be found in infected tissues [2, 3]. Interestingly, both morphological forms have benefits for surviving in different conditions. Yeast form* C. albicans* cells are tolerated by the host's immune system, while a hyphal form triggers specific host responses [4]. Yet, yeast form* C. albicans* was found to be engulfed more rapidly by macrophages than the hyphal form [5]. Recently, a mouse model for vulvovaginal candidiasis (VVC) was established that highlighted the requirement of pattern recognition receptors (PRRs) for the induction of S100 alarmins [6].

While in healthy individuals the immune system generally controls a yeast to hypha transition, in immunocompromised patients, such as human immunodeficiency virus- (HIV-) infected individuals or patients receiving massive antibiotic treatment or chemotherapy,* C. albicans* can develop hyphae leading to a wide variety of superficial, mucosal, and systemic infections [7, 8]. Moreover,* C. albicans* may cause genitourinary infection, such as balanitis in men and VVC in woman [2]. In accordance, a great number of women have been diagnosed with VVC caused by* C. albicans* at least once in their life-time. While VVC mostly occurs in immunocompetent women, pregnant or diabetic women can suffer from recurrent VVC, which seriously deteriorates the quality of life [2].

Intense work has been done to characterize the response of* C. albicans* in different host-pathogen systems. Phagocytosis by macrophages first induces a shift to a nutrient poor condition by upregulating gluconeogenesis and fatty acid beta-oxidation and simultaneously downregulating translation. The expression of hyphae specific genes later enables hyphal growth thereby facilitating escape from macrophages [9]. Reconstituted human oral epithelium induced hyphae formation of* C. albicans*, which was followed by invasion via active and passive penetration of hyphae into cells. Transcript analysis showed that filamentous growth was induced in response to neutral pH, nonglucose carbon sources, and nitrosative stress [10].

Despite recent advances made in our understanding of disease pathogenesis caused by* C. albicans*, little is known about the mechanisms that underlie hyphal transition in response to contact with human vaginal epithelial cells.

Here we used the vaginal epithelial cell line PK E6/E7 cocultured with live* C. albicans* to model VVC and performed transcriptome sequencing in order to identify genes differentially expressed in* C. albicans* upon yeast to hyphae transition. Our results show that, at the transcriptome level, starvation, temperature, and CO_2_ concentration were all able to induce hyphal growth of* C. albicans*, both in the absence and in the presence of vaginal epithelial cells. Strikingly, the* N*-acetyl-D-glucosamine (GlcNAc) biosynthesis of* C. albicans* was specifically activated solely in the presence of human vaginal epithelial cells. Hence, our results suggest that the GlcNAc pathway has an important role in the virulence of* C. albicans* upon vulvovaginal candidiasis.

## 2. Materials and Methods

### 2.1. Strains and Growth Conditions

*C. albicans* clinical isolate SC5314 was grown on YPD medium (10 g/L yeast extract, 20 g/L bacto peptone, 20 g/L dextrose, and 2% agar) at 30°C, cultured under standard conditions until logarithmic phase, and then counted with a haemocytometer.

### 2.2. Cell Culturing

The immortalized human vaginal epithelial cell line (VECL) PK E6/E7 [12] was cultured in serum-free complete keratinocyte medium (CKM) supplemented with 5 ng/mL recombinant epidermal growth factor, 50 *μ*g/mL bovine pituitary extract, L-glutamine, and antibiotic/antimycotic solution (all from Life Technologies) in a CO_2_ thermostat at 37°C [13]. Cells at 60–70% confluency were used in subsequent experiments.

### 2.3. Fungal-Mammalian Cell Coculture

A total of 10^5^ PK E6/E7 VECL cells were seeded in 6-well plates and incubated for 24 hours in serum-free CKM. At 24 hours prior to infection with* C. albicans*, the medium was changed to serum-free CKM (pH 8.0) without antibiotic/antimycotic solution. Fungal cells were collected in log phase, washed three times with CKM, and then resuspended in complete CKM without antibiotic/antimycotic solution to eliminate farnesol. In order to induce hyphal growth, plates were incubated in a CO_2_ thermostat at 37°C (control hyphae). Fungal cells, treated the same way, were added to wells with a multiplicity of infection (MOI) of 3 : 1 to infect PK E6/E7 VECL. Yeast control cells were harvested at 0 hour time point. Plates were incubated for 3 hours in a humidified atmosphere containing 5% CO_2_ at 37°C; fungal cells rapidly switch to filamentous growth under such circumstances. Ten randomly chosen fields of view were used to count* C. albicans* hyphae penetrating into vaginal epithelial cells.

### 2.4. *C. albicans* Adherence Assay

PK E6/E7 VECL cells were grown in 6-well plates until confluency was reached (>90%). The* hxk1Δ* mutant and the parental strain (DIC185) [14] were grown on YPD plates for 24 hours. A total of 1 × 10^5^ cells resuspended in CKM were used to infect vaginal epithelial cells for 90 minutes. Supernatant was then aspirated and the wells were washed two times with 1× PBS. The monolayers with attached* C. albicans* were fixed by 3.7% (v/v) paraformaldehyde in PBS. Quantitation of* C. albicans* adherence was performed by light microscopy at a 25x magnification. Ten randomly chosen fields of view covered with epithelial cells were counted. Significance was calculated with a two-sample *t*-test and a* p* value of less than 0.05 was considered significant. Experiments were performed with at least three biological replicates.

### 2.5. Viability Test

The effect of* C. albicans* infection onto the viability of PK E6/E6 VECL cells was performed by Real-Time Cell Analysis (RTCA; ACEA Biosciences), as described previously [15–17]. Briefly, 10^4^ PK E6/E7 cells per well were seeded in 96-well E-plates (ACEA Biosciences) in which the bottoms of the wells were covered with micro electrodes and the epithelial cells were allowed to attach to the bottom of the wells and grow for 3 days. Cells were then treated with 2 × 10^3^, 5 × 10^3^, 1 × 10^4^, and 2 × 10^4^ *C. albicans hxk1Δ* or DIC185 cells/well. Triton-X (Sigma) treatment was used as a positive control to kill the vaginal epithelial cells. Subsequent real-time measurements of impedance were done with the xCELLigence System RTCA HT Instrument (ACEA Biosciences); the impedance was monitored every 10 minutes. The cell index at each time point was defined as (*R*_*n*_ − *R*_*b*_)/15, where *R*_*n*_ is the cell-electrode impedance of the well when it contains cells and *R*_*b*_ is the background impedance of the well with the medium alone. The cell index (CI) was normalized to the latest time point before the treatment of each group (CIn/CI before treatment) or presented as percent of nontreated control group [(CIn/CI average of control group) × 100]. CI values reflect cell number, adherence, cell growth, and health. Data are presented as means ± standard deviation (SD). Statistical significance between treatment groups was determined using one-way and two-way ANOVA following Bonferroni multiple comparison posttest (GraphPad Prism 5.0; GraphPad Software). Experiments were repeated three times; the number of biological replicates varied between 3 and 6.

### 2.6. Total RNA Isolation

Cells were harvested and resuspended in 400 *μ*L AE buffer (50 mM NaOAc, 10 mM EDTA); 40 *μ*L 10% SDS and 440 *μ*L of phenol were added and the samples vortexed. The mix was incubated at 65°C for 10 min and frozen in liquid nitrogen. After thawing, the samples were centrifuged with 10000 ×g for 2 minutes; the upper phase was extracted with phenol-chloroform and precipitated with 1/10th volume of 3 M NaOAc and 2.5 × volume of 96% ice cold EtOH. Finally, the samples were centrifuged (150000 RPM, 15 min), the supernatant was discarded, and the pellet was washed with 70% EtOH and resuspended in TE buffer (10 mM Tris-HCl, 1 mM EDTA, pH 7.5). RNA quality and quantity measurements were performed on Bioanalyzer (Agilent Technologies) and Qubit (Life Technologies).

### 2.7. High Throughput Sequencing

Whole transcriptome sequencing was performed as described previously [18]. Briefly, total RNA samples from three biological replicates were pooled in equimolar concentrations and processed using the SOLiD total RNA-Seq Kit (Life Technologies), according to the manufacturer's instructions. For this, 5 *μ*g of pooled RNA was DNaseI treated and fragmented using RNaseIII; the eukaryotic ribosomal RNA was depleted prior to fragmentation using RiboMinus Eukaryote Kit for RNA-Seq and RiboMinus Concentration Module (Life Technologies). Next, the 50–200 nt RNA fraction was size-selected, sequencing adaptors were ligated, and the templates were reverse-transcribed using ArrayScript reverse transcriptase. The cDNA library was purified with Qiagen MinElute PCR Purification Kit (Qiagen) and size-selected on a 6% TBE-Urea denaturing polyacrylamide gel. The 150–250 nt cDNA fraction was amplified using AmpliTaq polymerase and purified by AmPureXP Beads (Agencourt). The concentration of each library was determined using the SOLID Library TaqMan Quantitation Kit (Life Technologies). Each library was clonally amplified on SOLiD P1 DNA Beads by emulsion PCR (ePCR). Emulsions were broken with butanol and ePCR beads enriched for template-positive beads by hybridization with magnetic enrichment beads. Template-enriched beads were extended at the 3′ end in the presence of terminal transferase and 3′ bead linker. Beads with the clonally amplified DNA were deposited onto SOLiD flowchip and sequenced on SOLiD V4 instrument using the 50 + 35-base paired-end sequencing chemistry.

### 2.8. Bioinformatic Analysis

Bioinformatic analysis of the whole transcriptome sequencing was performed in colour space using Genomics Workbench (CLC Bio). Raw sequencing data were trimmed by removal of low quality, short sequences so that only 50 and 35 nucleotide long sequences were used in further analysis. Sequences were mapped in a strand specific way onto the* C. albicans* SC5314 genome assembly 19 reference genome [19], using default parameters except for the following: minimum length 50% and minimum similarity 80% with the unspecific match limit set to 10. Normalized gene expression was calculated using the “scaling” normalization method [20].

### 2.9. Statistical Analysis of Differential Gene Expression

Differentially expressed genes from the RNA-Seq output were determined using the R package DEGSeq. The software calculates significance with an MA-plot method for RNA-Seq data without biological replicates. Gene expression was considered significantly different between two conditions if the false discovery rate (FDR) corrected probability (*p*) value was less than 0.05 [21] and the absolute fold change value was more than 2.0 [22].

### 2.10. Quantitative Reverse Transcriptase Polymerase Chain Reaction (QRT-PCR)

cDNA was synthesized from at least 100 ng of high quality (RIN > 8.5) total RNA by using the High Capacity RNA-to-cDNA Kit (Life Technologies) according to the manufacturer's instructions. SybrGreen technology-based real-time quantitative PCR was used to quantify the relative abundance of the selected mRNAs; primer sets are listed in Table S3. As controls, we used reaction mixtures without cDNA. Relative expression of the given gene in the yeast-like form was set to 1 and the expression in control hyphae (*C. albicans* cells grown in CKM) and hyphae developed in the presence of PK E6/E7 vaginal epithelial cells was calculated by comparing the values to the yeast-like form. All measurements were performed in duplicate with at least three biological replicates. The ratio of each mRNA relative to the 18S rRNA was calculated using the 2^−ΔΔCT^ method; all the data are presented as mean ± standard deviation.

### 2.11. Data Availability

Gene Expression Omnibus (GEO) archive of the three sequenced libraries was deposited in NCBI's GEO Archive at http://www.ncbi.nlm.nih.gov/geo under accession GSE54694.

## 3. Results

### 3.1. Vaginal Epithelial Cell-*C. albicans* Coculture as a Model of Vulvovaginal Infection

**Figure 1:**
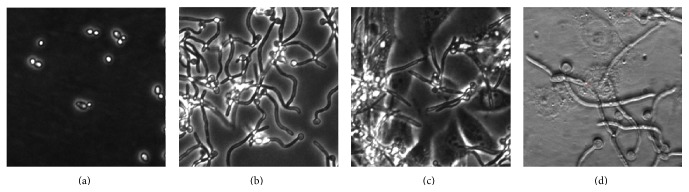
Microscopic analysis of* C. albicans* hyphal growth* in vitro*. Yeast form of* C. albicans* (a);* C. albicans* develops hyphae in CKM (b) and in the presence of PK E6/E7 vaginal epithelial cells (c).* C. albicans* hyphae penetrate into PK E6/E7 VECL cells (d).

Infection of epithelial cells by* C. albicans* requires adhesion of yeast form cells to the surface of epithelium, a process that aids in inducing a morphological switch resulting in hypha formation. We used the immortalized PK E6/E7 vaginal epithelial cell line (PK E6/E7 VECL) [12] cultured in complete, serum-free keratinocyte medium (CKM), containing 1,0 g/L (0.1 v/w%, or 5.6 mM) glucose, and infected them with* C. albicans* SC5314 yeast form cells (Figure 1(a)). Since we aimed to monitor the primary effect of human cells onto the hyphae formation, we sampled the cells at 3 hours postinfection (pi). As control,* C. albicans* cells were cultured in complete keratinocyte medium (CKM) without serum (the culture media of PK E6/E7 VECL) for 3 hours. Microscopic examination showed that at this time point* C. albicans* cells adhering to the surface of the culture chamber developed hyphae (control hyphae) even in the absence of serum (Figure 1(b)). Notably, when cocultured, conditions changed drastically, such as CO_2_ concentration, temperature, and being neutral to alkaline pH, all of which are known to strongly induce the morphological transition of* C. albicans* [11]. Thus,* C. albicans* cells adhered to the surface of PK E6/E7 VECL and developed hyphae (Figures 1(c) and 1(d)), but only approximately 5% of hyphae penetrated into epithelial cells (Figure 1(d)). Importantly, control hyphae and hyphae developed in the presence of PK E6/E7 VECL could not be distinguished in terms of the timing of the morphological switch, rate of hyphae development, or length of hyphae (Figures 1(b) and 1(c)).

### 3.2. Primary Analysis of Transcriptome Data

To study the early and specific molecular events occurring upon hyphae formation in the absence or presence of vaginal epithelial cells, global transcriptome changes of* C. albicans* cells were monitored using RNA-Seq. To do this, transcriptomes of yeast form* C. albicans* (C.a.0 h),* C. albicans* forming hyphae in the absence of host cells (control hyphae; C.a.3 h), and* C. albicans* hyphae induced by PK E6/E7 VECL (PK + C.a.3 h) were sequenced on SOLiD System (Table 1). Reads were aligned to the* C. albicans* SC5314 genome (assembly 19) and normalized gene expression changes calculated as described in Materials and Methods. Comparison of gene expression was carried out with the help of DEGSeq software and a difference in gene expression change above 2.0-fold and false discovery rate (FDR) less than 0.05 were considered significant.

Pairwise comparisons showed that when compared to the yeast-like form the expression of 1283 and 2537 genes was significantly altered when* C. albicans* cells developed hyphae in a serum-free medium (control hyphae) or when* C. albicans* developed hyphae in the presence of PK E6/E7 vaginal epithelial cells, respectively (Table 2). Interestingly, we identified 1574 genes with altered expression when comparing the two different hyphal growth conditions: hyphae developed in the presence of human cells as compared to control hyphae (Table 2 and Table S1).

RNA-Seq data allowed us to identify 384 genes showing significantly higher expression in both C.a.3 h and PK + C.a.3 h compared to C.a.0 h samples without significant expression change between C.a.3 h and PK + C.a.3 h samples (Table S2). These genes might be considered as effector genes of hyphae formation as a response of culturing* C. albicans* in serum-free CKM. Thus, our results show that 376 genes exhibited altered expression in both C.a.3 h and PK + C.a.3 h samples with significant difference in their expression between these two samples. Moreover, the expression of 1205* C. albicans* genes was exclusively altered in the PK + C.a.3 h samples (Table S2): these genes may play a role in virulence of* C. albicans* after contact with vaginal epithelial cells.

### 3.3. Validation of RNA-Seq Data by Quantitative Real-Time PCR (QRT-PCR)

**Figure 2:**
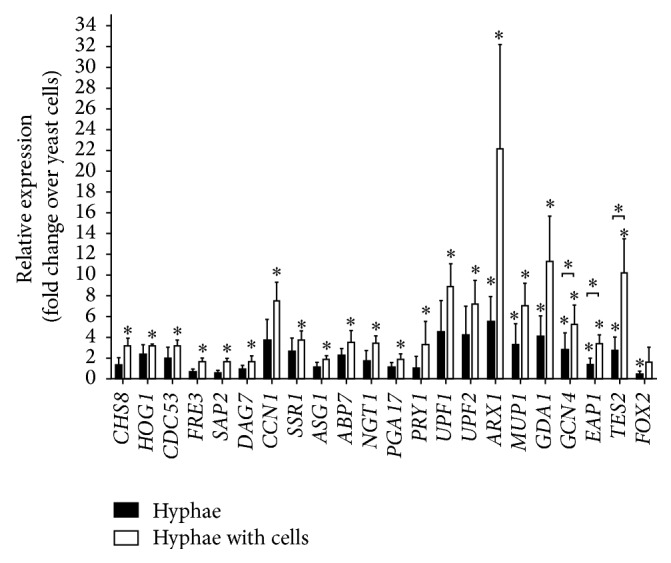
QRT-PCR validation of RNA-Seq results. The relative gene expression of selected genes shows altered expression upon hyphae development as compared to yeast-like growth. Black and open bars represent control hyphae (*C. albicans* cells grown in CKM) and hyphae developed in the presence of PK E6/E7 vaginal epithelial cells, respectively. The ratio of each mRNA relative to the 18S rRNA was calculated using the 2^−ΔΔCT^ method. Data are representative of 3 or more independent experiments and are presented as mean ± standard deviation (SD). The significance of differences between sets of data was determined by two-sample *t*-test; ^*∗*^ *p* < 0.05. For gene names, please see Table S1 (see Supplementary Material available online at http://dx.doi.org/10.1155/2015/398045).

QRT-PCR analyses were performed to validate the expression pattern of 22 genes (Figure 2): this gene set includes representatives of all the identified expression patterns (see above). The QRT-PCR analysis showed that, as compared to yeast-like form cells, 15 genes (among others,* CHS8*,* HOG1*, and* CDC53*) were indeed significantly upregulated in PK + C.a.3 h, but not in C.a.3 h samples. In addition,* ARX1*,* MUP1*, and* GDA1* were upregulated in both PK + C.a.3 h and C.a.3 h samples with no significant expression difference in between these two samples.* GCN4*,* EAP1*, and* TES2* were upregulated in both PK + C.a.3 h and C.a.3 h samples, but with significantly higher expression in PK + C.a.3 h. Finally, expression of* FOX2* was significantly downregulated in both C.a.3 h and PK + C.a.3 h samples. Importantly, the results of QRT-PCR analysis are in complete agreement with the RNA-Seq expression data (Figure 2 and Table S2).

### 3.4. Functional Analysis of RNA-Seq Data

#### 3.4.1. Carbohydrate Metabolism and Fatty Acid Oxidation

The expression of several genes involved in carbohydrate metabolism changed in both hyphal forms of* C. albicans *when compared to the yeast form. Generally, expressions of mRNAs encoding enzymes involved in glycolysis were downregulated. Specifically, expression of* PFK1*, an enzyme of the rate-limiting step of glycolysis, was downregulated, while* FBP1* encoding the rate-limiting enzyme of gluconeogenesis was significantly upregulated both in control hyphae and in hyphae induced by PK E6/E7 VECL (2.6-fold and 5.7-fold, resp.). In accordance,* CDC19*, catalyzing citrate synthesis from phosphoenolpyruvate, was significantly downregulated in both hyphal forms (−3.3-fold and −2.6-fold, resp.). In contrast, expression of* PCK1* catalyzing the conversion of oxaloacetate to phosphoenolpyruvate, thus fueling gluconeogenesis, was markedly induced (15.7-fold) in the control hypha and only moderately upregulated (5.3-fold) in the hypha induced by the PK E6/E7 VECL (Table S1).

Gluconeogenesis is fueled by the glyoxylate cycle with oxaloacetate. Key enzymes of the glyoxylate cycle, such as* ICL1* and* MLS1*, were markedly upregulated both in the control hypha and hypha induced by PK E6/E7 VECL. Other enzymes involved in the glyoxylate cycle, but shared with the tricarboxylic acid (TCA) cycle, like* ACO1*,* ACO2*, and* MDH*, were also strongly induced in control hyphae and hyphae induced by PK E6/E7 VECLs (Figure S1).

Glucose deprivation induces fatty acid beta-oxidation resulting in acetyl coenzyme A (acetyl-CoA) production [9]. The expression of the two isoenzymes for acetyl-CoA C-acyltransferase (*POT1*,* FOX3*) and* FOX2* was downregulated, while the expression of* POT1-2* was slightly induced (Figure S2). In contrast, expression of one of the isoenzymes for acetyl-CoA C-acyltransferase (*POX1*) and one isoform of the long chain fatty acid-CoA ligase (*FAT1*) was significantly upregulated in both the control hyphae and hyphae induced by PK E6/E7 VECL. These data suggest that enzymes responsible for the first two steps of the beta-oxidation pathway were upregulated upon hyphal growth indicating that beta-oxidation might be responsible for the production of acetyl-CoA (Figure S2).

Downregulation of glycolysis and simultaneous upregulation of gluconeogenesis, the glyoxylate cycle and fatty acid beta-oxidation indicates a shift from nutrient rich to nutrient poor condition in our* in vitro* system. Thus, application of the serum-free CKM medium may induce yeast to hyphae morphogenesis via starvation irrespective of vaginal epithelial cells.

#### 3.4.2. Analysis of Signal Transduction Pathways Involved in Hyphal Morphogenesis

**Figure 3:**
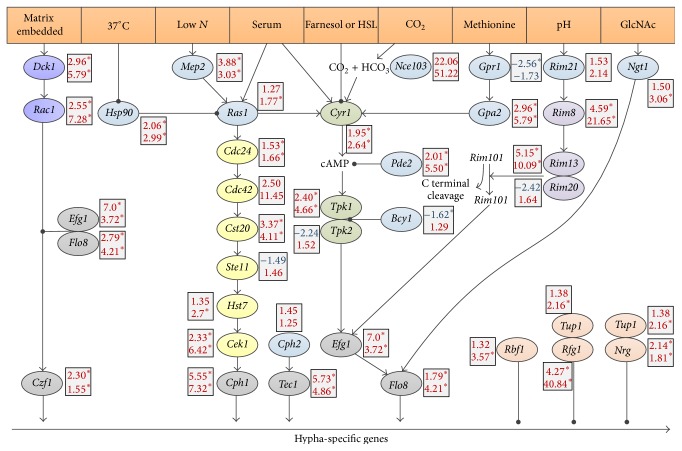
Comparison of gene expression of signal transduction pathways and their components* in vitro*. Figure was redrawn from Sudbery [11]; genes are shown with gene names. Upper and lower numbers show fold change difference of expression between the C.a.3 h and the C.a.0 h or the PK + C.a.3 h and the C.a.0 h samples, respectively. Significant changes in gene expression are marked by asterisk; values depicted in red indicate upregulation; in contrast, those in blue show downregulation.

Low energy culturing conditions induced strong hyphal morphogenesis of* C. albicans* either with or without PK E6/E7 VECL (Figure 1). Thus, we sought to determine if other signal transduction pathways leading to hyphal morphogenesis are responding to these conditions at the level of transcription. We found that the* DCK1-RAC1* pathway, known to be required for filamentous growth in a matrix embedded microenvironment, is upregulated upon hyphal growth without cells (3.0- and 2.6-fold, resp.) and further upregulated in hyphae induced by PK E6/E7 VECL (5.8- and 7.3-fold, resp.) (Figure 3). Notably, the expression of* CZF1* was only upregulated in C.a.3 h but not in PK + C.a.3 h samples (Figure 3). Moreover, expression of* MEP2* transducing low nitrogen signal towards RAS1 was also enhanced during hyphal growth (Figure 3).

Neither the expression of the* RAS1*, which is known as a signal integrator, nor the expressions of* CDC24* or* STE11* altered significantly. However, a significantly elevated expression was detected for* CDC42*,* CST20*,* CEK1*, and* CPH1* both in the C.a.3 h and in PK + C.a.3 h samples (Figure 3), while* HST7* expression increased only in the PK + C.a.3 h sample. The adenylyl cyclase (CYR1) pathway in* C. albicans* also functions as a signal integrator for different environmental conditions and is regulated directly by farnesol, CO_2_, glucose and methionine concentration, RAS1, and serum [11] (Figure 3).* CYR1* expression was induced only in the PK + C.a.3 h sample, but the expression of the components of the CYR1 pathway, such as* GPA2*,* PDE2*,* TPK1*,* EFG1*, and* FLO8*, was upregulated in both control hyphae and hyphae induced by PK E6/E7 VECL. Of note, we identified a significant induction in the expression of* EFG1*, encoding a transcriptional activator having a major effect on the induction of the hyphal specific genes, both in C.a.3 h and in PK + C.a.3 h samples (7.0- and 3.72-fold, resp.) (Figure 3). Consistently,* FLO8* expression was also upregulated in both C.a.3 h and PK + C.a.3 h samples. Our results indicate that morphological transition and upregulation of master transcription factors* EFG1* and* FLO8* occur in parallel, irrespective of epithelial cells.

We also monitored the expression of major repressors of hyphae specific genes. Slight but significant increases in expression were observed for* RBF1*,* TUP1*, and* NRG1* in response to VECL. Interestingly, we found a robust increase in the expression of* RFG1* in response to both control hyphae and hyphae induced by PK E6/E7 VECL. Both* NRG1* and* RFG1* are known to repress transcription of hyphae specific genes, along with* TUP1* in response to serum and temperature [23]. It is thus reasonable to suggest that the ratio of transcriptional activators and repressors is fine-tuning the expression of the hyphae specific genes (Figure 3).

GlcNAc is known to induce hyphal morphogenesis [24] and white opaque switching [25, 26] in* C. albicans*. Interestingly, we found that the* NGT1* gene representing the transporter gene in the* N*-acetyl-D-glucosamine transporter was solely upregulated in hyphae induced by PK E6/E7 VECL (3.1-fold), but not in control hyphae (1.5-fold), indicating the specificity of this response to epithelial cells (Figure 3).

We have identified a parallel upregulation of several hyphal induction pathways at the level of transcription both in the control hyphae and in hyphae induced by PK E6/E7. Therefore, other parameters, such as glucose concentration and pH, were measured which may also lead to hyphal induction in* C. albicans*. We found that pH reduced from 8.0 to 7.6 ± 0.04 in control hyphae and to 7.6 ± 0.02, when VECL were also present (Table 3). Glucose concentration was also reduced from 5.2 mM to 4.58 mM and 4.6 mM in control hyphae and hyphae developed in the presence of VECL, respectively (Table 3). These data show that both pH and glucose concentration changed in a similar way and extent in our* in vitro* system. These values are, however, still in the range in which a yeast to hyphae transition in* C. albicans* is strongly induced [27, 28].

### 3.5. Expression Analysis of Genes Involved in GlcNAc Metabolism

**Figure 4:**
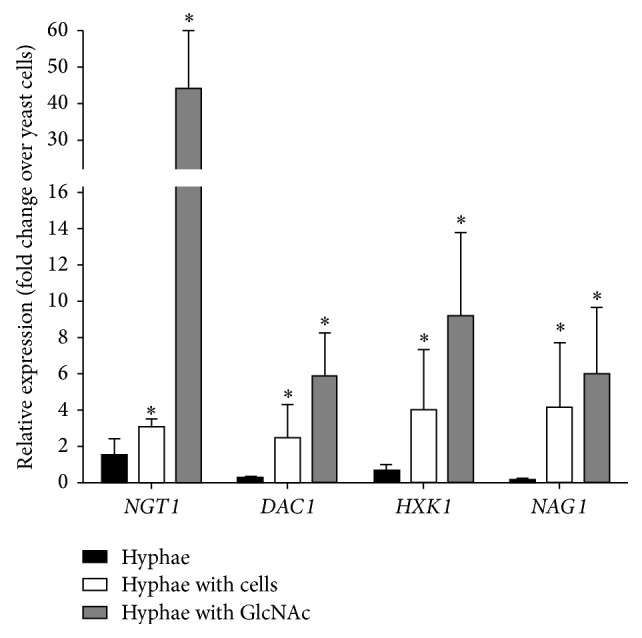
QRT-PCR analysis of the expression of GlcNAc catabolism genes. The relative gene expression of selected genes shows altered expression upon hyphae development as compared to yeast-like growth. First column (black bars) represents* C. albicans* cells grown in CKM (control hyphae); the second (open) and third (gray) columns stand for hyphae developed in the presence of PK E6/E7 vaginal epithelial cells and* C. albicans* cells grown in CKM + 10 mM of GlcNAc, respectively. The ratio of each mRNA relative to the 18S rRNA was calculated using the 2^−ΔΔCT^ method. Data are representative of 3 independent experiments and are presented as mean ± standard deviation (SD). The significance of differences between sets of data was determined by two-sample *t*-test; ^*∗*^ *p* < 0.05.

As GlcNAc induces hyphal morphogenesis in* C. albicans* [29], we sought to monitor the expression of GlcNAc catabolic genes in our* in vitro* model. Since the RNA-Seq experiment did not provide sufficient number of unique reads for statistical analysis of this group (data not shown), the expression of a number of GlcNAc catabolic genes was tested by QRT-PCR. For this, the following conditions were used: control hyphae, hyphae induced by vaginal epithelial cells, and control hyphae supplemented with 10 mM of GlcNAc. Expressions of GlcNAc deacetylase (*DAC1*), hexokinase 1 (*HXK1*), and GlcNAc deaminase (*NAG1*) were all repressed in control hyphae as compared to the yeast form* C. albicans*; the expression of* NGT1* remained unaltered (Figure 4). Lack of induction of these three genes may be due to the fact that these cells were cultured in a mammalian culture medium containing glucose. These results are in agreement with a previous report, which showed that glucose did not significantly induce the expression of GlcNAc catabolism genes [14]. Furthermore, our results showed that the expressions of all four genes (*NGT1*,* DAC1*,* HXK1*, and* NAG1*) genes involved in GlcNAc catabolism were all significantly upregulated in the hyphae induced by vaginal epithelial cells and upon GlcNAc induction (Figure 4). Moreover, GlcNAc could be formed by the enzymatic effect of the* C. albicans* HEX1 protein that is able to liberate GlcNAc from the cell wall glycoproteins of* C. albicans* [30]. For this reason the expression pattern of* HEX1* was determined. According to our RNA-Seq data, the expression of* HEX1* increased solely, but not significantly (1.8-fold, Table S1), in response to vaginal epithelial cells, which could provide a plausible explanation for the specific expression of the GlcNAc catabolism genes. Finally, administration of 10 mM GlcNAc caused definite expression of the GlcNAc catabolic genes that is probably due to the high concentration of GlcNAc (Figure 4).

GlcNAc is also fueling chitin synthesis by producing UDP-GlcNAc [29, 31]. Therefore, we also monitored the expression of genes involved in the GlcNAc to UDP-GlcNAc conversion (*AGM1*,* UAP1*) and some of the chitin synthases (*CHS*), which require UDP-GlcNAc to produce chitin [29]. Our RNA-Seq data showed that expression of* AGM1*,* UAP1*,* CHS2*,* CHS3*, and* CHS7* robustly increased both in control hyphae and in the presence of vaginal epithelial cells (Table 4) indicating that the expression of these genes is likely hyphae specific.

### 3.6. GlcNAc Is Involved in the Adherence of* C. albicans* to Vaginal Epithelial Cells

**Figure 5:**
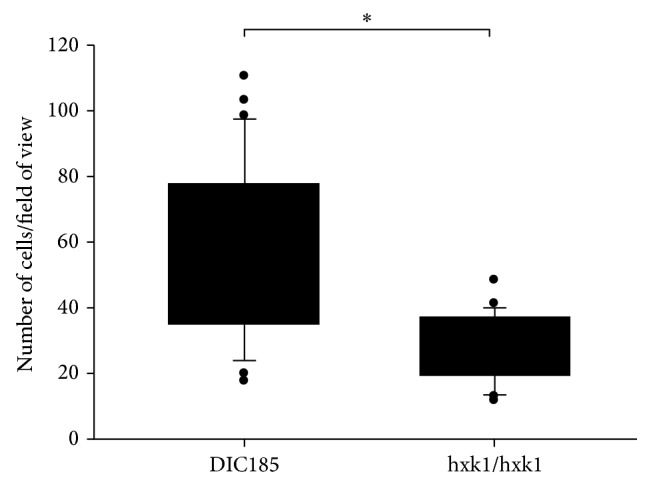
Adherence of the* C. albicans* parental strain DIC185 and* hxk1Δ* mutant to PK E6/E7 vaginal epithelial cells. The *y*-axis represents the number of* C. albicans* cells that remained adhered. The significance of differences between sets of data was determined by two-sample *t*-test; ^*∗*^ *p* < 0.05.

We next sought to determine the importance of the GlcNAc metabolic pathway in our* in vitro* system. Taking into account genes involved in the GlcNAc catabolic pathway many deletion mutants, such as* ngt1Δ*,* hxk1Δ*,* nag1Δ*, and* dac1Δ*, have a similar phenotype [14]; therefore they could all be excellent candidates for using them in functional assays. However,* nag1Δ* and* dac1Δ* mutants could not grow on glucose if the medium contained GlcNAc [14]; hence we have chosen to use a* hxk1Δ* mutant strain in our subsequent experiments. To determine if the GlcNAc pathway is involved in the attachment of* C. albicans* to the surface of vaginal epithelial cells, we carried out an adherence assay. Monolayers of PK E6/E7 vaginal epithelial cells were treated with 3 × 10^5^ yeast form* C. albicans* parental (DIC185) and mutant (*hxk1Δ*) strains. After 90 min of contact, which is enough for* C. albicans* cells to form hyphae, nonadhered cells were washed away and the numbers of adherent* C. albicans* cells were counted. Our results showed that significantly less* hxk1Δ* mutant remained attached to the surface of the PK E6/E7 cells compared to the DIC185 parental strain (Figure 5). This data indicates the importance of* HXK1* gene and therefore the GlcNAc pathway in the adherence of* C. albicans* to vaginal epithelial cells.

### 3.7. The GlcNAc Pathway Is a Virulence Factor in the* In Vitro* Vulvovaginal Candidiasis Model

**Figure 6:**
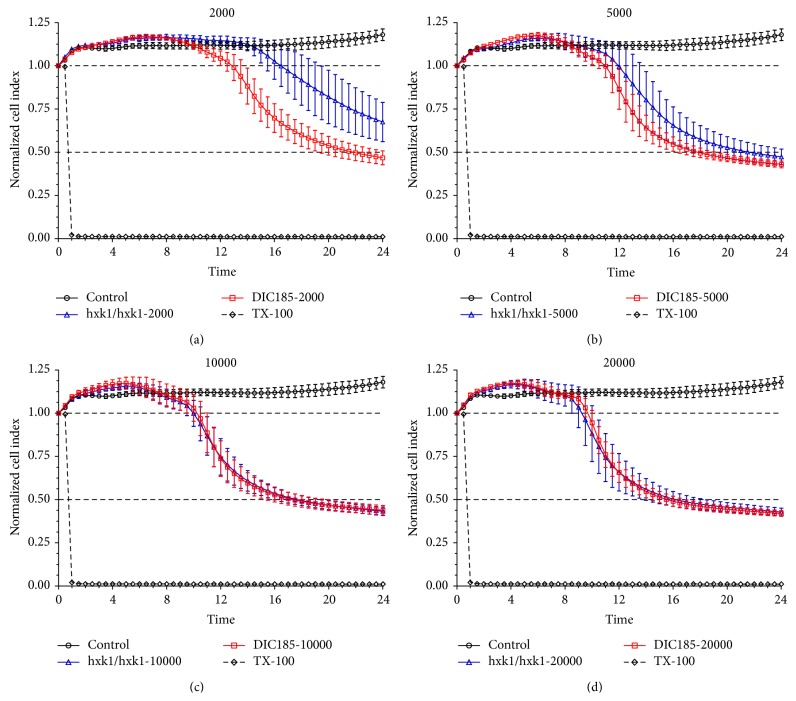
Effect of* C. albicans* parental (DIC185) and* hxk1Δ* mutant (hxk1/hxk1) strains on the viability of PK E6/E7 vaginal epithelial cells. Cell index (CI) was measured using the RTCA method by xCELLigence System. CI was plotted as a function of time postinfection. Different numbers of yeast form* C. albicans* were used as inoculum: (a) 2000 cells/well, (b) 5000 cells/well, (c) 10000 cells/well, and (d) 20000 cells/well.

**Figure 7:**
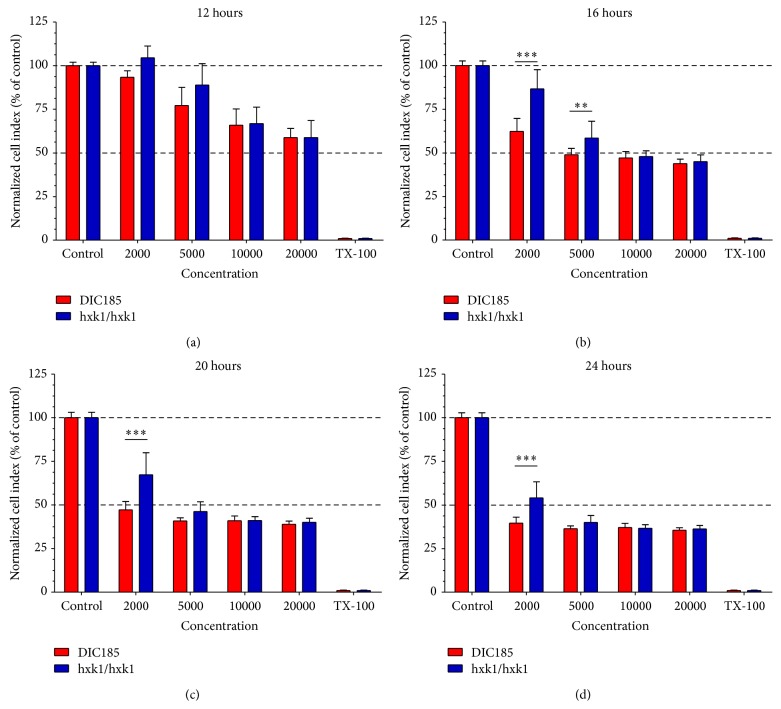
Statistical analysis of the RTCA viability test. Cell indexes reflecting viability of PK E6/E7 vaginal epithelial cells infected with the same number of* C. albicans* parental (DIC185) and* hxk1Δ* mutant (hxk1/hxk1) strains were compared. Cell indexes were plotted as a function of inoculum size. Changes were considered statistically significant at *p* < 0.05 (^*∗*^); *p* < 0.01 (^*∗∗*^); and *p* < 0.001 (^*∗∗∗*^).

Adherence of* C. albicans* to epithelial cells is followed by invasion of the surface [32]. Given the strong correlation between adhesion, invasion, and virulence of* C. albicans*, we next tested if the lack of the GlcNAc pathway, which has an important role in the adherence, also affects virulence of* C. albicans *in our* in vitro* system. For this, we used an RTCA assay, which provides real-time, quantitative information about the number of the living, attached cells by measuring electrode impedance. Vaginal epithelial cells were treated with different numbers of yeast form* C. albicans* parental (DIC185) or mutant (*hxk1Δ*) strains and the impedance was measured for 24 hours and the data converted to cell index (CI) (as described in Section 2.5). Microscopic examination showed that both* hxk1Δ* and DIC185 strains behaved similarly in terms of germ tube formation and germ tube length in all inoculum concentrations during the experiment (data not shown). Our results show that the CI index of nontreated cells slightly increased, while cells treated with Triton X-100 rapidly detached the plate surface because of massive cell lysis (Figure 6). When PK E6/E7 VECL cells were infected with low numbers (2000 and 5000) of* C. albicans*, the* hxk1Δ* mutant exhibited lower cytotoxicity as compared to the parental strain DIC185 considered as wild type (Figures 6(a) and 6(b)). When the number of infecting* C. albicans* cells was increased (10000 and 20000 cells) the* hxk1Δ* mutant no longer exhibited a reduced cytotoxic effect (Figures 6(c) and 6(d)). We also determined that the cytotoxic effect exhibited by wild type* C. albicans* increased with the cell number used for infection (Figures 6 and 7). Finally, when 2000 yeast cells were used for infection, the CI of vaginal epithelial cells infected with the* hxk1Δ* mutant was significantly higher at 16, 20, and 24 hours postinfection as compared to the control DIC185 strain (Figures 7(b), 7(c), and 7(d), resp.). At increasing* C. albicans* cell numbers used for infection, we only measured a significantly higher cell index of the* hxk1Δ* mutant at 16 hours postinfection (5000 cells; Figure 7(b)).

These results show that the* hxk1* deletion attenuates virulence of* C. albicans* in our vulvovaginal candidiasis* in vitro* system, and this attenuation depends on the yeast cell number.

## 4. Discussion

### 4.1. Evaluation of Our* In Vitro* Vulvovaginal Candidiasis Model

Secretions of the female genital tract keep the epithelial surface of the vagina moist. Moreover, the lactic acid concentration of the vaginal fluid creates a pH of approximately 4.5 [33]. However, lactic acid concentration and pH similar to that of the vaginal fluid greatly inhibited cell division and germ tube formation of* C. albicans* [34, 35]. In contrast, we now show that, in the presence of vaginal epithelial cells cultured in CKM (pH 8.0) containing glucose at 5.6 mM concentration, rapid, synchronous, and robust hyphal morphogenesis of* C. albicans* could be induced. Thus, our samples were homogenous to get valid gene expression data. Therefore we believe that our* in vitro* conditions resemble the invasive growth of* C. albicans* into epithelial cells. However, hyphae induction also occurred with the same trend and extent, when yeast form* C. albicans* cells were incubated in serum-free CKM. In line with our results, 0.1% (w/v) glucose (5.6 mM) strongly induced hypha development of* C. albicans* on solid media [28]. Moreover, our RNA-Seq data showed strong upregulation of the gluconeogenesis, glyoxylate cycle, and fatty acid beta-oxidation pathways in both the control hyphae and hyphae developed in the presence of VECL. Consistently, our RNA-Seq results are in complete agreement with an earlier report, in which microarray analysis of phagocytosed* C. albicans* cells revealed the upregulation of the glucose starvation related metabolic pathways, such as gluconeogenesis, glyoxylate cycle, and fatty acid beta-oxidation [9]. Strikingly,* C. albicans* strains isolated from diabetic individuals suffering from vulvovaginal candidiasis showed high isocitrate-lyase and malate-synthase enzymatic activities [36]. Interestingly, glucose concentration in blood is very similar to that of CKM and remarkably, the vaginal fluid contains ~5.2 mM glucose as a final concentration [37]. CKM is thus a low glucose medium and, in this respect, it is similar to vaginal fluid. Hence, starvation to glucose may be one factor that drives the yeast to hyphae transition of* C. albicans* in our* in vitro* system.

*C. albicans* is highly adapted to humans; thus a wide range of environmental conditions induces hyphal morphogenesis [11, 38]. In our* in vitro* system, the expression of* DCK1*-*RAC1*,* RAS1* and most of the components of the* CYR1* driven signal transduction pathways were upregulated in* C. albicans* both in the presence and in the absence of vaginal epithelial cells.* RAC1* and its activator* DCK1* are required for filamentous growth of* C. albicans* in matrix embedded conditions [39].* RAS1* with* CDC24* positively regulates the MAPK pathway necessary for the expression of hyphae specific genes in* C. albicans* [38].* RAS1* is able to sense signals generated by low nitrogen, serum and temperature and also activates Cyr1 [11, 38]. The cAMP-PKA pathway plays a very important role in* C. albicans* filamentation [40]. An elevated level of cAMP, which is catalyzed by the adenylate cyclase CYR1, is required for yeast to hyphae transition [41]. cAMP activates PKA (TPK1 and 2), which positively regulates EFG1, the major transcriptional activator of hyphae specific genes [38]. CYR1 is activated by serum, RAS1 transmitted signals, and up to 5% of CO_2_. Although RAS1 transduces low nitrogen, serum, and temperature generated signals to CYR1, while CYR1 is directly activated by CO_2_ signal [42].

### 4.2. The Role of GlcNAc in the Virulence of* C. albicans*

Microenvironment massively induced many components of several different signal transduction pathways leading to morphological transitions in* C. albicans* both in the control hyphae and in hyphae developed in response to vaginal epithelial cells. Strikingly, the GlcNAc induced* NGT1* was markedly induced exclusively in response to vaginal epithelial cells (Figures 2 and 4). We also found that the* C. albicans hxk1Δ* mutant adhered to the surface of vaginal epithelial cells at a significantly lower level (Figure 5), and* hxk1Δ* mutant exhibits reduced cytotoxicity as compared to the wild type* C. albicans* strain (Figures 6 and 7). The human extracellular matrix contains a significant amount of GlcNAc [29].* C. albicans* infection can cause massive tissue damage, mostly via active penetration of growing* C. albicans* hyphae, leading to the death of epithelial cells [32]. In agreement with a recent review [29], GlcNAc released from the extracellular matrix of human cells during membrane remodeling might explain the induction of* C. albicans* GlcNAc catabolic genes, such as,* NGT1*,* HXK1*,* NAG1*, and* DAC1* (Figure 4), by vaginal epithelial cells. Alternatively, the HEX1 protein of* C. albicans* secreted into the medium is able to liberate GlcNAc from carbohydrate side-chains of cell wall proteins of* C. albicans*, but not by mammalian cells [43], which in turn could trigger the expression of the GlcNAc catabolic genes [30]. This explanation was further supported by our results demonstrating that the expression of* HEX1* was increased in response to vaginal epithelial cells.

GlcNAc is transported by the N-acetylglucosamine transporter (NGT1) [44] and then phosphorylated by HXK1 in* C. albicans* [14, 31] with GlcNAc-6-PO_4_ fueling the N-acetyl-D-glucosamine biosynthesis. We anticipate that the GlcNAc inducible N-acetyl-D-glucosamine biosynthesis could not be responsible for the attenuated virulence, since it is contributing to direct GlcNAc-6-PO_4_ to glycolysis [29]. We rather propose that the GlcNAc anabolic pathway is partly responsible for the reduced adherence and cytotoxicity of the* hxk1Δ* mutant within our* in vitro* vulvovaginal candidiasis system. Adherence to the surface of epithelial and endothelial cells and penetration of hyphae into these cells are important virulence factors contributing to the pathogenesis of* C. albicans* [45]. Moreover, the inner layer of the cell wall of* C. albicans* consists of polymers of *β*-(1,3)-glucan, *β*-(1,6)-glucan, and GlcNAc (chitin). This scaffold binds cell wall proteins, glycosylphosphatidylinositol- (GPI-) anchor-dependent cell wall proteins (GPI-CwPs), which play an important role in the adherence of* C. albicans* to the epithelial cells [11]. The GlcNAc biosynthesis plays a key role in chitin biosynthesis by producing UDP-GlcNAc and is also involved in N-linked glycosylation [29]. Although enzymes for GlcNAc biosynthesis and chitin synthases showed hyphae specific (or low glucose inducible) expression at the mRNA level in our* in vitro* vulvovaginal candidiasis system, the lack of feeding this pathway with GlcNAc-6-PO_4_ in the* hxk1Δ* mutant might cause reduced chitin content for effective adherence [46]. Alternatively, GlcNAc not phosphorylated in the* hxk1Δ* mutant accumulates at high level, which might inhibit enzymes using UDP-GlcNAc as substrate, such as chitin biosynthesis and GPI-anchor synthesis. Consistently, higher chitin content was measured in the hyphal form of* C. albicans* than in the yeast form [47] and that further links the attenuated virulence phenotype of* hxk1Δ* mutant to the cell wall of* C. albicans*. This is in agreement with a recent report showing that microevolution of the nonfilamentous* Candida glabrata* with macrophages results in a mutant having higher chitin synthase activity, pseudohyphal growth, and stronger virulence [48]. Moreover, the* hxk1Δ* mutant* C. albicans* showed increased sensitivity to the competitive chitin synthesis inhibitor Nikkomycin Z [49], indicating the significant involvement of hexokinase 1 protein in cell wall synthesis [31]. Strikingly, Nikkomycin Z caused reduced adherence of* C. albicans* to the surface of buccal epithelial cells [50]. Finally, the fact that the* C. albicans* chitin synthase 7 (CHS7) mutant strain has a similar phenotype to that of* hxk1Δ* mutant, such as sensitivity to Nikkomycin Z and reduced virulence [51], implies that GlcNAc biosynthesis in* C. albicans* acts as a virulence factor in our* in vitro* vulvovaginal candidiasis system.

## 5. Conclusion

Taken together, our serum-free* in vitro* system modeling the vaginal microenvironment was able to induce* C. albicans* hyphal morphogenesis via different signal transduction pathways. By using this model combined with RNA sequencing, we demonstrated that hyphal morphogenesis could be triggered by several signal transduction pathways. However, the GlcNAc biosynthesis of* C. albicans* is highly dependent on the presence of vaginal epithelial cells. Hence, our results highlight the importance of the GlcNAc biosynthesis in the virulence of* C. albicans* in vulvovaginal candidiasis.

## Supplementary Material

RNA seq data was analyzed and differentially expressed genes between the C.a.3h vs. C.a.0h, PK+C.a.3h vs. C.a.0h and PK+C.a.3h vs. C.a.3h samples were listed. Since the hyphal form of C. albicans is pathogenic, genes with altered expression upon hyphal growth were collected. Our gene expression analysis revealed that the expression of genes involved in the glyoxylate cycle and fatty acid beta-oxidation in C. albicans were upregulated.

## Figures and Tables

**Table 1:** Number of sequence tags generated on SOLiD V4. Note that 50 + 35-base paired-end sequencing chemistry was applied.

Samples	Total number of reads	Reads mapped in pairs	Reads mapped in broken pairs
C.a.0 h	24,855,550	8,130,060	6,048,926
C.a.3 h	28,460,342	8,242,582	7,610,411
PK + C.a.3 h	61,576,468	5,635,126	5,500,084

**Table 2:** Number of differentially expressed genes in pairwise comparisons; gene expression change above 2.0-fold and with FDR corrected *p* value of less than 0.05 was considered significant.

Pairwise comparison	Number of differentially expressed genes
C.a.3 h/C.a.0 h	1283
PK + C.a.3 h/C.a.0 h	2537
PK + C.a.3 h/C.a.3 h	1574
Common	689

**Table 3:** Glucose concentration and pH change during yeast to hyphae transition of *C. albicans* irrespective of the presence of vaginal epithelial cells. Samples were taken at starting point (0 h) and at end point (3 h) of the experiment.

	pH (0 h)	pH (3 h)	Glucose (0 h)	Glucose (3 h)
C.a.3 h/C.a.0 h	8,01 (±0)	7,59 (±0,04)	5,6 (±0.1) mM	4,58 (±0,39) mM
PK + C.a.3 h/C.a.0 h	8,01 (±0)	7,55 (±0,02)	5.6 (±0.09) mM	4,60 (±0,23) mM

**Table 4:** Expression of genes required for converting GlcNAc to UDP-GlcNAc and chitin synthases in our *in vitro* system according to RNA-Seq data. Numbers represent fold changes.

	C.a.3 h/C.a.0 h	PK + C.a.3 h/C.a.0 h
AGM1	3.83	4.41
UAP1	7.62	26.14
CHS1	n.d.	n.d.
CHS2	1.74	3.93
CHS3	4.56	8.51
CHS7	3.82	13.77
