# Supplementary figures and images for: The Absence of N-Acetyl-D-glucosamine Causes Attenuation of Virulence of Candida albicans upon Interaction with Vaginal Epithelial Cells In Vitro
Source: Biomed Res Int. 2015 Aug 20;2015:398045. doi: 10.1155/2015/398045 (PMC4558442; doi:10.1155/2015/398045)

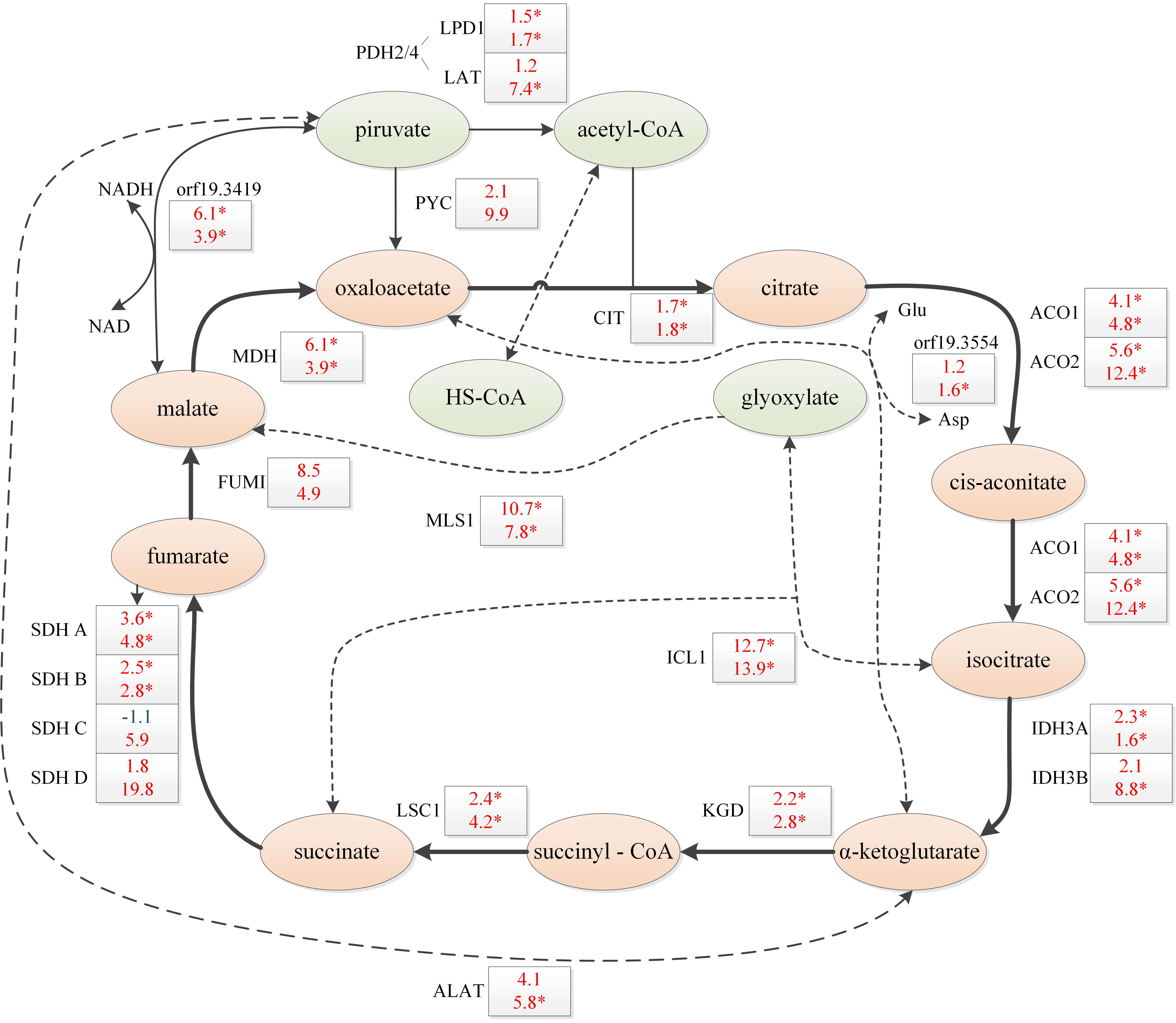

Supplement: Supplementary file 1 — RNA seq data was analyzed and differentially expressed genes between the C.a.3h vs. C.a.0h, PK+C.a.3h vs. C.a.0h and PK+C.a.3h vs. C.a.3h samples were listed. Since the hyphal form of C. albicans is pathogenic, genes with altered expression upon hyphal growth were collected. Our gene expression analysis revealed that the expression of genes involved in the glyoxylate cycle and fatty acid beta-oxidation in C. albicans were upregulated. [file 398045.f1.zip › sup/BMRI Manczinger et al figure S1.tif]

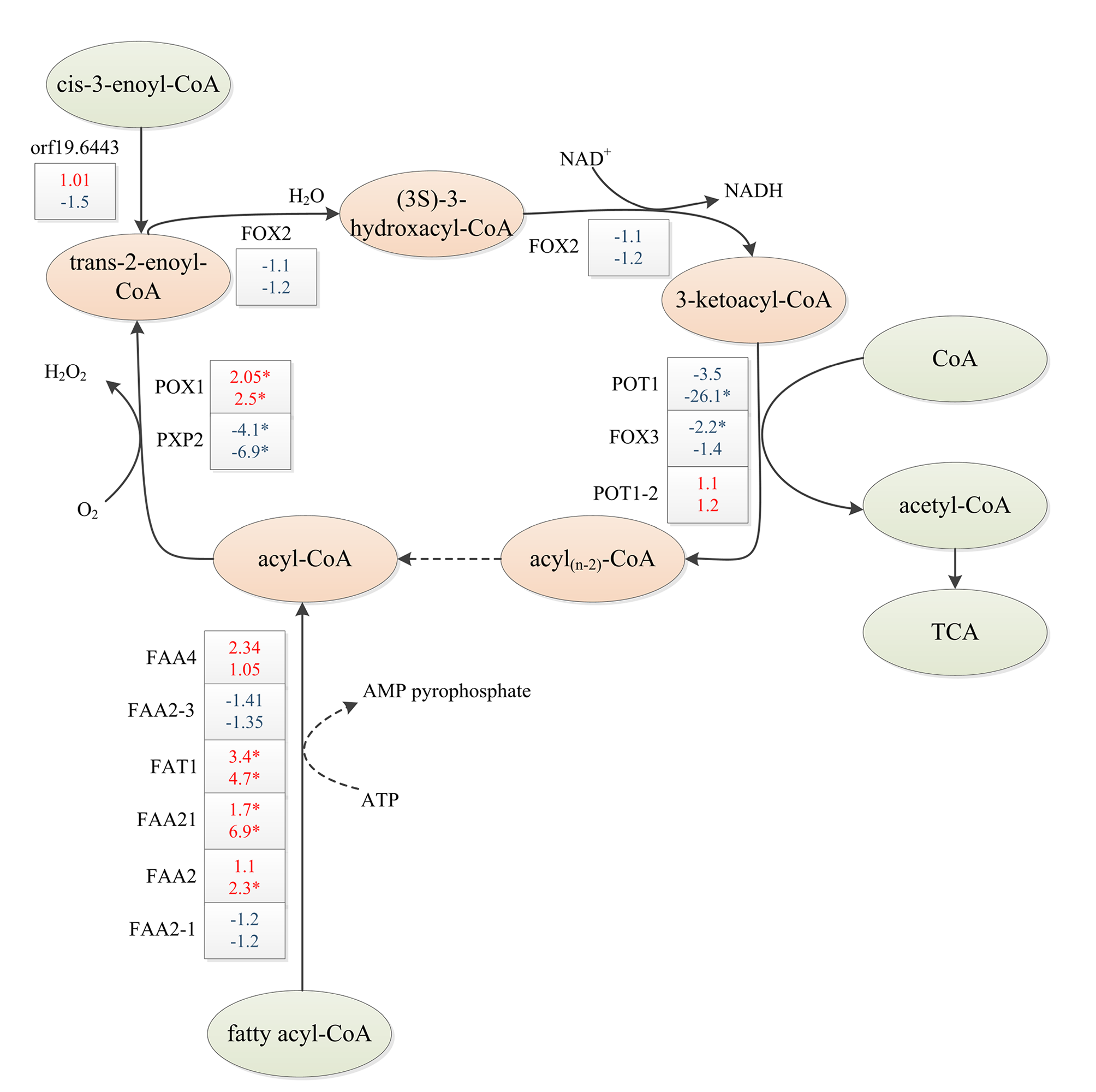

Supplement: Supplementary file 1 — RNA seq data was analyzed and differentially expressed genes between the C.a.3h vs. C.a.0h, PK+C.a.3h vs. C.a.0h and PK+C.a.3h vs. C.a.3h samples were listed. Since the hyphal form of C. albicans is pathogenic, genes with altered expression upon hyphal growth were collected. Our gene expression analysis revealed that the expression of genes involved in the glyoxylate cycle and fatty acid beta-oxidation in C. albicans were upregulated. [file 398045.f1.zip › sup/BMRI Manczinger et al Figure S2.tif]
